# New Advanced Liquid Crystalline Materials Bearing Bis-Azomethine as Central Spacer

**DOI:** 10.3390/polym14061256

**Published:** 2022-03-21

**Authors:** Fowzia S. Alamro, Hoda A. Ahmed, Noha S. Bedowr, Muna S. Khushaim, Mohamed A. El-atawy

**Affiliations:** 1 Department of Chemistry, College of Science, Princess Nourah bint Abdulrahman University, P.O. Box 84428, Riyadh 11671, Saudi Arabia; fsalamro@pnu.edu.sa; 2 Department of Chemistry, Faculty of Science, Cairo University, Cairo 12613, Egypt; 3 Chemistry Department, College of Sciences, Taibah University, Yanbu 30799, Saudi Arabia; nbedowr@taibahu.edu.sa; 4 Department of Physics, Faculty of Science, Taibah University, P.O. Box 30002, Al-Madina 41447, Saudi Arabia; mkhushaim@taibahu.edu.sa; 5 Nanotechonolgy Center, Taibah University, P.O. Box 30002, Al-Madina 41447, Saudi Arabia; 6 Chemistry Department, Faculty of Science, Alexandria University, P.O. Box 426 Ibrahemia, Alexandria 21321, Egypt

**Keywords:** bis-azomethine liquid crystals, mesomorphic properties, geometrical structure, DFT

## Abstract

In this study, a homologous series of novel liquid crystalline compounds bearing the bis-azomethine central linkage (–CH=N-N=CH–), namely ((1E,1′E)-hydrazine-1,2-diylidenebis(methanylylidene))bis(4,1-phenylene) dialkanoate (**I*n***), was synthesized, and the mesophase and thermal properties were investigated theoretically and experimentally. The molecular structures of the prepared compounds were determined using elemental analysis, NMR, and FT-IR spectroscopy. The mesophase transitions were detected by differential scanning calorimetry (DSC), and the mesophases were identified using polarized optical microscopy (POM). The results indicated that the derivative with the shortest length (**I*5***) was purely nematogenic, while the other homologues (**I*9*** and **I*15***) possessed SmC mesophases. The optimal geometrical structures of the investigated group were derived theoretically. The estimated results demonstrated that all homologues were mesomorphic, and their type depended on the length of the terminal chains. Computations based on density functional theory (DFT) were used to explain the experimental data. The calculated dipole moment, polarizability, thermal energy, and molecular electrostatic potential all showed that it was possible to predict the mesophase type and stability, which varied according to the size of the molecule.

## 1. Introduction

Schiff bases are compounds that contain an azomethine group. They are produced from condensation of the carbonyl group of aldehyde or ketone with primary amines. Compared to the original aldehydes or ketones, they possess extra donor sites, thus enabling them to be elastic and more flexible [1,2,3,4]. In addition, they display good physical and chemical properties, including high surface area, purity, durability, and permeability. Due to their importance to bioinorganic systems and their use in catalysis, anticorrosion, and various industrial areas, Schiff bases have also gained significant attention among chemists [5,6,7,8,9,10].

The terminal polar group has a significant effect on the mesomeric properties of derivatives containing Schiff bases. Azo and azomethine are attractive connecting groups for designing new mesomorphic structures because of their photoactivity under UV irradiation [11,12,13,14,15,16,17,18,19,20]. The length of the liquid crystal molecular conjugation can be increased to enhance birefringence. However, the photostability and solubility of LCs with high birefringence should be considered [15]. The resulting mesomorphic properties of such molecules clearly reflect this aspect. Although there are changes in the polarity and orientation of dipole moments, there is little change in the molecular structure. As a result, a small change in molecular geometry enhances the optical properties, resulting in different mesophase behavior. The molecules tend to align in a parallel pattern as the length of the terminal substituent increases [19]. The twist–bend nematic and heliconical phases are similarly influenced by the length of the terminal chains [20,21].

Interestingly, computational assumption for newly designed materials [22,23,24,25,26,27] has attracted some attention. Stimulated information regarding the orbital energies of molecules and molecular geometries of liquid crystalline molecules is necessary to give a wide range of optical properties. Because of its higher performance and consistent computational output, density functional theory (DFT) has lately emerged as a promising technique. Many researchers have recently become interested in material design based on computational prediction [22,23,24,25,26,28,29,30,31,32]. The mutual interaction of several optical factors demands stimulated information regarding molecular orbital energies and LC molecular geometries. Furthermore, DFT is a strong tool that quickly provides insight into the properties of molecules.

The goal of this research was to synthesize new bis-Schiff base liquid crystals with two symmetrical terminal substituents to yield the homologous series ((1E,1′E)-hydrazine-1,2-diylidenebis(methanylylidene))bis(4,1-phenylene) dialkanoate (**I*n***). All homologues were designed to be symmetrical around the central linkage (–CH=N-N=CH–). The phenyl moieties were coupled to the terminal by alkanoate chains containing 5, 9, and 15 carbons. Density functional theory (DFT) was used to analyze the experimental mesomorphic properties in terms of predicted parameters as well as the impact of the linking bis-azomethine (–CH=N-N=CH–) groups and terminal alkanoate chain length.


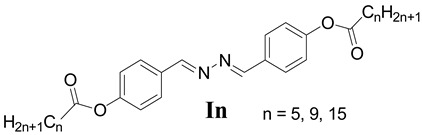


## 2. Experimental

### Synthesis

**Scheme 1:**
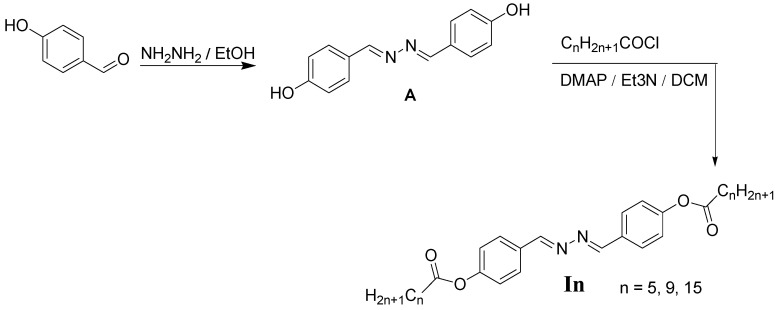
Synthesis of ((1E,1′E)-hydrazine-1,2-diylidenebis(methanylylidene))bis(4,1-phenylene) dialkanoate (**I*n***).

The liquid crystals **I*n*** were established as follows (Scheme 1):

Details and analyses of the synthesis of (**A**) [33] and **I*n*** are given in Supplementary Materials.

## 3. Results and Discussion

### 3.1. Chemistry

The bis-Schiff base **I*n*** series was prepared in a two-step process. First, direct condensation was carried out between 4-hydroxybenzaldehyde and hydrazine hydrate in molar ratio (2:1) to afford diphenol (**A**), namely, 4,4′-((1E,1′E)-hydrazine-1,2-diylidenebis(methanylylidene))diphenol. Then, subsequent esterification of the diphenol using acid chloride (hexanoyl chloride, decanoyl chloride, and palmitoyl chloride) was conducted in the presence of triethylamine/DMAP as base catalyst and dichloromethane as solvent to yield the desired Schiff bases. Infrared spectrum of the **I*n*** compounds exhibited bands in the 1750–1770 cm^−1^ region, which can be attributed to the absorption characteristic of carbonyl ester stretching. **I*n*** showed the characteristic infrared absorption bands for C=N stretching at 1600–1650 cm^−1^. Moreover, as a prototype, the proton NMR (DMSO-*d6*) of **I*9*** exhibited a singlet at δ 8.65 ppm due to the existence of azomethine protons. The presence of the aliphatic protons was confirmed by a group of signals in the 2.59–0.87 ppm range. The ^13^C-APT–NMR spectrum of **I*9*** exhibited three quaternary carbons at δ 172, 153.03, and 131.59 ppm. The most deshielded peak (172 ppm) was due to the carbonyl group. The other two peaks (153.03 and 131.59 ppm) were assigned to the ipso quaternary carbons of the benzene rings of the homologue **I*9***. This confirmed the same environment for the carbons and protons for the symmetrical structure of **I*9***.

### 3.2. Liquid Crystalline Investigations

**Figure 1:**
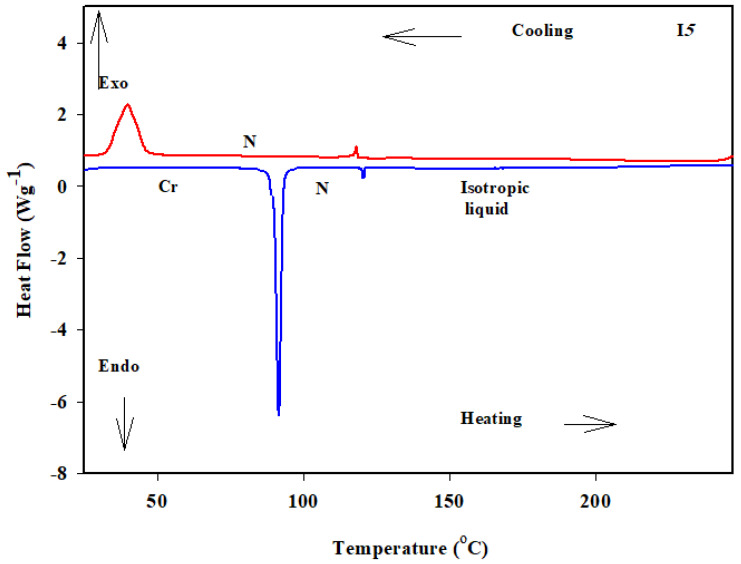
The second heating and cooling scan of compound **I*5*** provided DSC cycles with a heating rate of 10 °C min^−1^.

**Figure 2:**
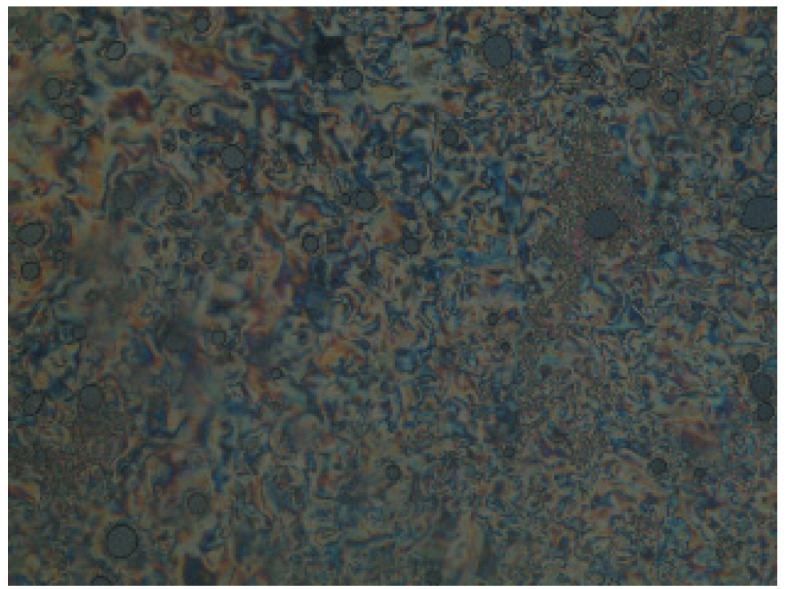
Texture of N phase observed under POM for compound **I*5***.

**Figure 3:**
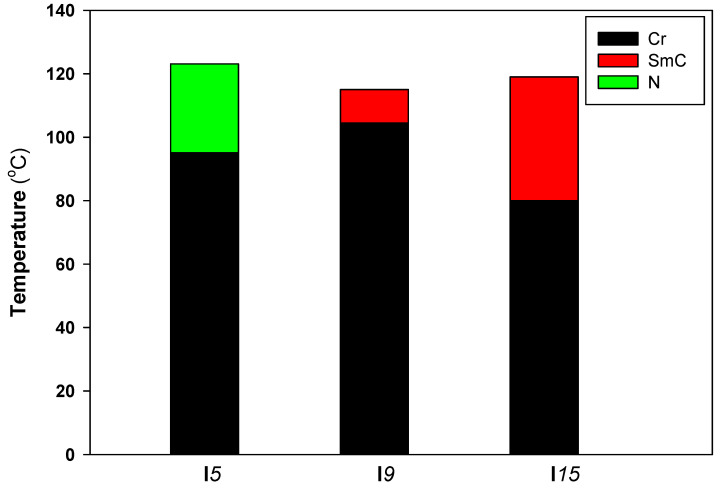
The effect of alkyl chain length on the mesophase behavior of the homologous **I*n*** series.

The synthesized **I*n*** series was investigated for its mesomorphic properties. Table 1 summarizes the results of the DSC experiments for transition temperatures and enthalpies. The stability of the mesophases of the synthesized homologues was investigated using DSC data from the second heating/cooling cycles. The second heating scan was utilized to record all the thermal characteristics. Figure 1 shows the DSC thermogram of the synthesized homologue **I*5*** as an example. On heating, the homologues revealed two endothermic peaks corresponding to the crystal-to-mesophase and mesophase-to-isotropic transitions, whereas two reversed exothermic peaks were observed on cooling, as shown in Figure 1. The DSC data was confirmed by the POM textures. POM image for the **I*5*** derivative is represented in Figure 2. All homologues were shown to possess enantiotropic monomorphic properties. The effect of the terminal alkyl chains on the mesomorphic behavior of the formed homologues was evaluated using a graphical representation of DSC temperature of transitions (Figure 3). Table 1 and Figure 3 reveal that the derivate with the shortest alkyl chain (**I*5***) exhibited pure nematic (N) phase, while the other two longer homologues (**I*9*** and **I*15***) were smectogenic and possessed the SmC mesophases. Generally, the mesomorphic behavior of any designed liquid crystalline molecular architecture is determined by the type of linking spacers, the length of the terminal chains, and the size of the attached substituents [34,35]. The melting transitions followed a random pattern, as seen in Table 1 and Figure 3. The homologue **I*15*** showed the lowest melting point (80.3 °C), while the homologue **I*9*** possessed the highest melting transition temperature (104.5 °C).

The polarity and/or polarizability of the mesogenic core of the molecule plays the most essential role in determining mesophase behavior. The homologue **I*5*** exhibited the highest mesophase stability (nematic stability = 122.6 °C), while the longer homologues **I*9*** and **I*15*** had smectic C stabilities of nearly 115.3 and 118.5 °C, respectively. As indicated by the results, the temperature range of the formed mesophases depended on the molecular anisotropy due to changes in the mesogenic core and geometry of terminal chains of the molecule. The geometrical characteristics, such as dipole moment, polarizability, and molecular shape of the produced **I*n*** homologues, had a significant impact on molecular association and led to enhanced formation of the N mesophase for the derivative with the shortest chain (**I*5***) and the SmC phases for the other longer homologues (**I*9*** and **I*15***).

Table 1 shows the normalized entropy changes (ΔS/R) of the synthesized **I*n*** compounds. The entropy value of the N transition was small due to the short length of the alkyl chain of compound **I*5***, while the entropy of SmC transitions for both **I*9*** and **I*15*** derivatives had higher magnitude than **I*5***. These results are in accordance with previous findings [34]. In addition, the terminal alkyl chains play an important role in multiconformational changes in the molecule [35].

### 3.3. Computational Calculations

#### 3.3.1. Thermal and Geometrical Parameters

**Figure 4:**
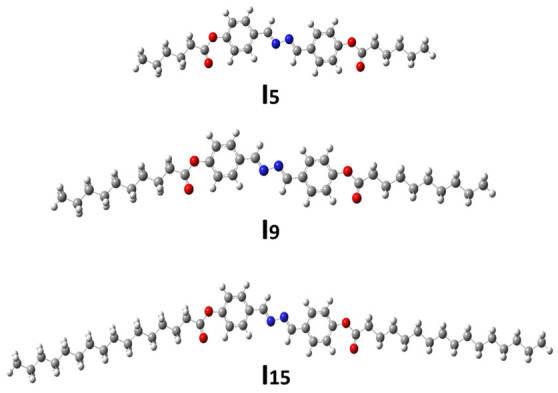
The optimized molecular geometries of the investigated **I*n*** compounds.

In the gas phase, the optimized molecular structures of the synthesized homologues (**I*n***) were investigated by applying the DFT/B3LYP methods using the 6–31G (d,p) basis [36,37] provided by GAUSSIAN 09W (Figure 4). The absence of imaginary frequency for all compounds demonstrated the stability of the optimized structures. The results of calculations revealed that all homologues were slightly linear, as shown in Figure 4. The magnitude of any system’s thermodynamic dynamic parameters, as well as its energy, is proportional to its length. This conclusion is supported by Table 2, which shows that the values of all estimated parameters increased as the length of the terminal alkyl chain increased [38,39,40,41,42,43,44].

#### 3.3.2. Frontier Molecular Orbitals (FMOs)

**Figure 5:**
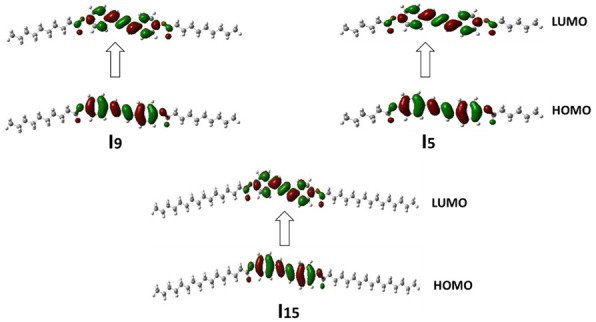
FMO’s estimated for the synthesized **I*n*** set.

The molecular orbital analysis showed that the frontier molecular orbitals (FMO’s) for the **I*n*** series predominantly consisted of *p* atomic orbitals, which corresponded to the π-bond of the two aromatic rings along with the π-bond of the linking azomethine groups, as shown in Figure 5. This would suggest that electronic transitions of these compounds was mainly of the π–π* type. Moreover, it was noticed that delocalization of the electron density was present in HOMO orbitals, whereas the LUMO orbitals showed extended delocalization on the carbonyl oxygen of the ester groups. Table 3 summarizes the resulting energies and energy gaps for the examined **I*n*** series. It was obvious that changing the length of the alkanoyl group (hexanoyl to hexadecanoyl group) led to increase in the energy of both HOMO and LUMO orbitals. Subsequently, the energy gaps (∆E) between FMO’s levels remained almost constant. Thus, the chemical reactivity of the **I*n*** series was quite similar for all the homologues.

#### 3.3.3. Molecular Electrostatic Potential (MEP)

**Figure 6:**
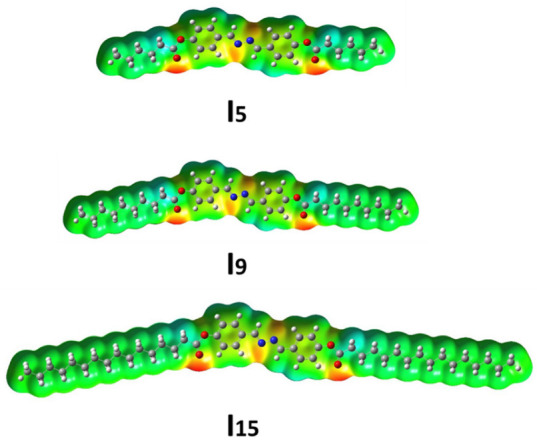
MEP estimated for the **I*n*** series.

The molecular electrostatic potential (MEP) is one of the most useful methods for determining whether the examined molecules have inter- or intramolecular interactions. The MEP of the current **I*n*** series is shown in Figure 6. MEP measures how attractive (red) or repulsive (blue) a proton placed at any position around the molecule is to any portion of the molecule. The MEP results revealed that the attracting portion (red) accumulated above the oxygen of acyl groups, indicating that these locations had a high electron density but low electrostatic potential. On the other hand, the blue region around the methylene groups and the adjacent phenyl hydrogen showed low electron density but strong electrostatic potential.

## 4. Conclusions

A novel bis-Schiff base homologues series, namely ((1E,1′E)-hydrazine-1,2-diylidenebis(methanylylidene))bis(4,1-phenylene) dialkanoate (**I*n***), was synthesized and examined via experimental and computational approaches. The thermal and mesomorphic properties were investigated using DSC and POM. All prepared compounds were found to be mesomorphic and possessing enantiotropic mesophases. The shortest homologue (**I*5***) exhibited pure N phase, while the other two homologues (**I*9*** and **I*15***) possessed purely SmC mesophases. Computational DFT study indicated that their thermal properties were length dependent.

## Figures and Tables

**Table 1:** Mesomorphic temperatures (°C), enthalpy (Δ***H***, kJ/mole), and entropy (Δ***S***/R) of transition for **I*n*** compounds.

Comp.	*T*_Cr–SmC_	∆*H*_Cr–SmC_	*T*_Cr–N_	∆*H*_Cr–N_	*T*_SmC–I_	∆*H*_SmC–I_	*T*_N–I_	∆*H*_N–I_	Δ*S/R*
**I*5***	-	-	95.1	47.3	-	-	122.6	1.9	0.58
**I*9***	104.5	42.1	-	-	115.3	2.3	-	-	0.71
**I*15***	80.3	43.7	-	-	118.5	2.1	-	-	0.64

Transitions of crystal to smectic C = _Cr–SmC_; crystal to Nematic = _Cr–N_; smectic C to isotropic liquid = _SmC–I_; nematic to isotropic liquid = _N–I_.

**Table 2:** Estimated thermal parameters for the investigated **I*n*** series.

Compound	ZPE (Kcal/Mol)	Thermal Energy (Kcal/Mol)	Enthalpy (Kcal/Mol)	Gibbs-Free Energy (Kcal/Mol)	Entropy (Cal mol.k)
**I*5***	336.356	357.302	357.894	289.931	227.950
**I*9***	479.617	507.397	507.990	422.723	285.985
**I*15***	694.540	732.568	733.161	621.898	373.176

**Table 3:** Thermal energy parameters for the investigated **I*n*** set.

Compound	Total Energy (Ha)	E_HOMO_ (ev)	E_luMO_ (ev)	∆E (ev)	Dipole Moment (D)	IE (ev)	EA (ev)	Polarizability Bohr^3^
**I*5***	−1419.805	−6.158	−2.239	3.919	2.9783	6.158	2.239	389.71
**I*9***	−1734.063	−6.155	−2.237	3.918	2.9384	6.155	2.237	486.30
**I*15***	−2205.449	−6.154	−2.236	3.918	2.9489	6.154	2.236	628.59

## Data Availability

The data presented in this study are available on request from the corresponding author.

## Supplementary Materials

The following supporting information can be downloaded at: https://www.mdpi.com/article/10.3390/polym14061256/s1, Figure S1: 1H-NMR of 4,4′-((1E,1′E)-hydrazine-1,2-diylidenebis(methanylylidene))diphenol; Figure S2: 13C-NMR of 4,4′-((1E,1′E)-hydrazine-1,2-diylidenebis(methanylylidene))diphenol; Figure S3: 1H-NMR of ((1E,1′E)-hydrazine-1,2-diylidenebis(methanylylidene))bis(4,1- phenylene) dihexanoate; Figure S4: 13C-NMR of ((1E,1′E)-hydrazine-1,2-diylidenebis(methanylylidene))bis(4,1- phenylene) dihexanoate; Figure S5: 1H-NMR of ((1E,1′E)-hydrazine-1,2-diylidenebis(methanylylidene))bis(4,1- phenylene) bis(decanoate); Figure S6: 13C-NMR of ((1E,1′E)-hydrazine-1,2-diylidenebis(methanylylidene))bis(4,1- phenylene) bis(decanoate); Figure S7: DSC cycles recorded from the second heating and cooling scan with heating rate 10 °C/min of compound I9; Figure S8: DSC cycles recorded from the second heating and cooling scan with heating rate 10 °C/min of compound I15.
