# Supplementary material for: New Advanced Liquid Crystalline Materials Bearing Bis-Azomethine as Central Spacer
Source: Polymers (Basel). 2022 Mar 21;14(6):1256. doi: 10.3390/polym14061256 (PMC8953589; doi:10.3390/polym14061256)
Supplement: Supplementary file 1 [file polymers-14-01256-s001.zip › polymers-1629826-supplementary.pdf]

Supplementary data

New advanced liquid crystalline materials bearing bis azomethine as central spacer

Fowzia S. Alamro[1], Hoda A. Ahmed [2,3,*], Noha S. Bedowr [3], Muna S. Khushaim [4,5], and Mohamed A. El-atawy [3,6,*]

[1]Department of Chemistry, College of Science, Princess Nourah bint Abdulrahman University, P.O. Box 84428, Riyadh 11671, Saudi Arabia, fsalamro@pnu.edu.sa
[2] Department of Chemistry, Faculty of Science, Cairo University, Cairo 12613, Egypt.
[3] Chemistry Department, College of Sciences, Yanbu, Taibah University, Yanbu 30799, Saudi Arabia; nbedowr@taibahu.edu.sa (N S.B)
[4]Department of Physics, Faculty of Science, Taibah University, P. Box: 30002, Al-Madina 41447, Saudi Arabia; mkhushaim@taibahu.edu.sa
[5] Nanotechonolgy Center, Taibah University, P. Box: 30002, Al-Madina 41447, Saudi Arabia
[6] Chemistry Department, Faculty of Science, Alexandria University, P.O. 426 Ibrahemia, Alexandria, 21321, Egypt

*Correspondence: author: Hoda A. Ahmed: ahoda@sci.cu.edu.eg and Mohamed A. El-atawy: mohamed.elatawi@alexu.edu.eg

1. *Materials*

Hexadecanoyl chloride, decanoyl chloride, hexanoyl chloride, 4-hydroxybenzaldehyde, and hydrazine hydrate were purchased from Sigma Aldrich (Germany). 4-dimethylaminopyridine (DMAP), Dichloromethane, and triethylamine were purchased from Aldrich (Wisconsin, USA).

2. Synthesis of 4,4'-((1E,1'E)-hydrazine-1,2 diylidenebis(methanylylidene))diphenol (**A**)

An ethanolic solution of p-hydroxy benzaldehyde (1.00 g, 8.20 mmol) was added dropwise to ethanolic solution of hydrazine monohydrate (0.200 g, 4.10 mmol) and resulting mixture was refluxed for 30 min. and then kept under stirring for 3-4 hours at room temperature till the starting materials are consumed, as indicated from TLC. A yellow precipitate formed, was washed with ethanol and diethyl ether and dried under vacuum.

3. Synthesis of ((1E,1'E)-hydrazine-1,2-diylidenebis(methanylylidene))bis(4,1-phenylene) dialkanoate, **I*n***

To a solution of the diphenol, 4,4'-((1E,1'E)-hydrazine-1,2 diylidenebis(methanylylidene))diphenol (**A**), 10 mmol in 30 mL of DCM, Triethylamine (Et3N) (20 mmol), dimethylaminopyridine (DMAP) (1 mmol) and the corresponding acyl chloride (12 mmol) were added at 0 °C and stirred. The reaction mixture was allowed to reach room temperature and was kept under stirring overnight at room temperature. The solvent was evaporated under reduced pressure, and the resultant oily residue dissolved in water and extracted with diethyl ether (3×30 mL). The combined organic phases were dried over anhydrous sodium sulfate (Na_2SO4) and filtered off and the solution evaporated till dryness under reduced pressure. Finally, purification by column chromatography afforded the desired liquid crystals, **I*n*.**

4,4'-((1E,1'E)-hydrazine-1,2 diylidenebis(methanylylidene))diphenol (**A**)

Yield: 93%; mp 107.0 °C, FTIR (ύ, cm^{-1}): 3400-3200 (OH), 2960, 2875 (CH_2 stretching), 1590 (C=N), 1610 (C=C). ^{1}H NMR (400 MHz, DMSO) δ 8.55 (s, 2H, CH=N), 7.70 (d, *J* = 8.3 Hz, 4H, Ar-H), 6.87 (d, *J* = 8.2 Hz, 4H, Ar-H). ^{13}C NMR (101 MHz, DMSO) δ 160.87 (CH), 160.75 (C), 130.58 (CH), 125.58 (C), 116.22 (CH). Elemental analyses: Found (Calc.): C, 69.99 (70.12); H, 5.03 (5.25); N, 11.66 (11.76).

2.2. ((1E,1'E)-hydrazine-1,2-diylidenebis(methanylylidene))bis(4,1-phenylene) dihexanoate, **I*5***

Yield: 94%; mp 95.0 °C, FTIR (ύ, cm^{-1}): 2950, 2885 (CH_2 stretching), 1763 (C=O), 1592 (C=N), 1600 (C=C). ^{1}H NMR (400 MHz, $CDCl_3$) δ 8.65 (s, 2H, CH=N), 7.87 (d, *J* = 8.6 Hz, 4H, Ar-H), 7.20 (d, *J* = 8.5 Hz, 4H, Ar-H), 2.59 (t, *J* = 7.5 Hz, 4H, 2 CH_2CO), 1.90 – 1.64 (m, 4H, 2CH_2), 1.41 (m, 8H, 4CH_2), 0.95 (t, *J* = 7.0 Hz, 6H, 2CH_3).^{13}C NMR (101 MHz, $CDCl_3$) δ 171.98 (CO), 161.12 (CH), 153.03 (C), 131.60 (C), 129.75 (CH), 122.10 (CH), 45.88 (CH_2), 34.38 (CH_2), 31.25 (CH_2), 24.57 (CH_2), 22.33 (CH_2), 13.93 (CH_3). Elemental analyses: Found (Calc.): C, 71.53 (71.36); H, 7.39 (7.18); N, 6.42 (6.53).

((1E,1'E)-hydrazine-1,2-diylidenebis(methanylylidene))bis(4,1-phenylene) didecanoate**, I*9***

Yield: 89 %; mp 104.0 °C, FTIR (ύ, cm^{-1}): 2970, 2870 (CH_2 stretching), 1765 (C=O), 1595 (C=N), 1610 (C=C). ^{1}H NMR (400 MHz, $CDCl_3$) δ 8.65 (s, 2H, CH=N),

7.87 (d, J = 8.6 Hz, 4H, Ar-H), 7.20 (d, J = 8.5 Hz, 4H, Ar-H), 2.59 (t, J = 7.5 Hz, 4H, $2CH_2$), 2.40 (t, J = 7.5 Hz, 4H, $2CH_2$), 1.82 – 1.73 (m, 4H, $2CH_2$), 1.63 – 1.52 (m, 6H, $3CH_2$), 1.28 (s, 14H, $7CH_2$), 0.93 – 0.87 (m, 6H, $2CH_3$). ^{13}C NMR (101 MHz, $CDCl_3$) δ 172.00 (CO), 161.13 (CH), 153.03 (C), 131.59 (C), 129.75 (CH), 122.10 (CH), 116.05 (CH), 42.83 (CH_2), 34.42 (CH_2), 31.87 (CH_2), 29.44 (CH_2), 29.43 (CH_2), 29.27 (CH_2), 29.26 (CH_2), 29.10 (CH_2), 24.89 (CH_2), 23.91 (CH_2), 22.67 (CH_2), 14.11(CH_3). Elemental analyses: Found (Calc.): C, 74.42 (74.38); H, 8.82 (9.10); N, 5.10 (5.25).

4. *Characterization*

Perkin-Elmer B25 (Perkin-Elmer, Inc., Shelton, CT USA) spectrophotometer was used for infrared spectra measurements. Varian EM 350L 400 MHz spectrometer (Oxford, UK) was used for recording ^{1}HNMR spectra with tetramethyl silane as internal standard in $CDCl_3$; the chemical shift values recorded as δ (ppm units). Thermo Scientific Flash 2000 CHS/O Elemental Analyzer, Milan, Italy was used for Elemental analyses. TA Instruments Co. (Q20 Differential Scanning Calorimeter, DSC; USA) was used for recording phase transitions. DSC calibration was carried using lead and indium melting temperature and enthalpy. Samples of 2–3 mg were used in aluminum pans for DSC investigation. The heating rate was 10°C/min in nitrogen gas as an inert atmosphere (30 ml/min). All transitions measured for the second heating scan. Transition temperatures for the prepared compounds were checked and phases identified by Polarized optical microscope (POM, Wild, Germany) attached with Mettler FP82HT hot stage. Absorption spectra of prepared films were recorded through Agilent Cary 5000 UV-Vis-NIR spectrophotometer. The steady state emission and time resolved decay spectra were recorded through Horiba delta flex TCSPC lifetime fluorometer by exciting the sample from Delta-diode 320 nm with peak width ±10 nm.

5. *Computational Method*

Gaussian 09 software [1] was used for DFT calculations for the studied compounds. DFT/B3LYP methods using 6-31G (*d,p*) basis set was selected for the calculations. The geometries were optimized by minimizing the energies with respect to all geometrical parameters without imposing any molecular symmetry constraints. The

structures of the optimized geometries had been drawn with Gauss View [2]. Also, calculations frequencies were carried out by the same level of theory. The frequency calculations showed that all structures were stationary points in the geometry optimization method with non-imaginary frequencies.

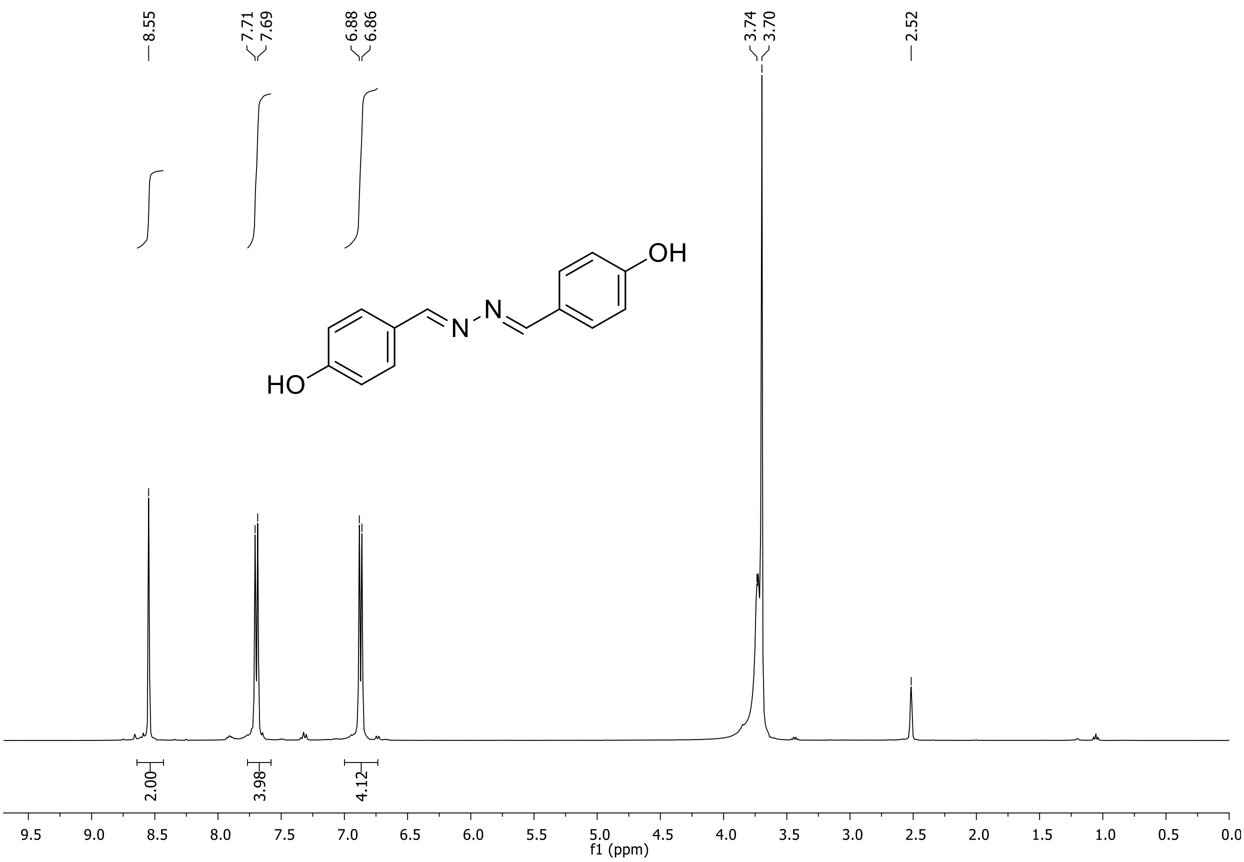

Figure S1. ^{1}H-NMR of 4,4'-((1E,1'E)-hydrazine-1,2-diylidenebis(methanylylidene))diphenol.

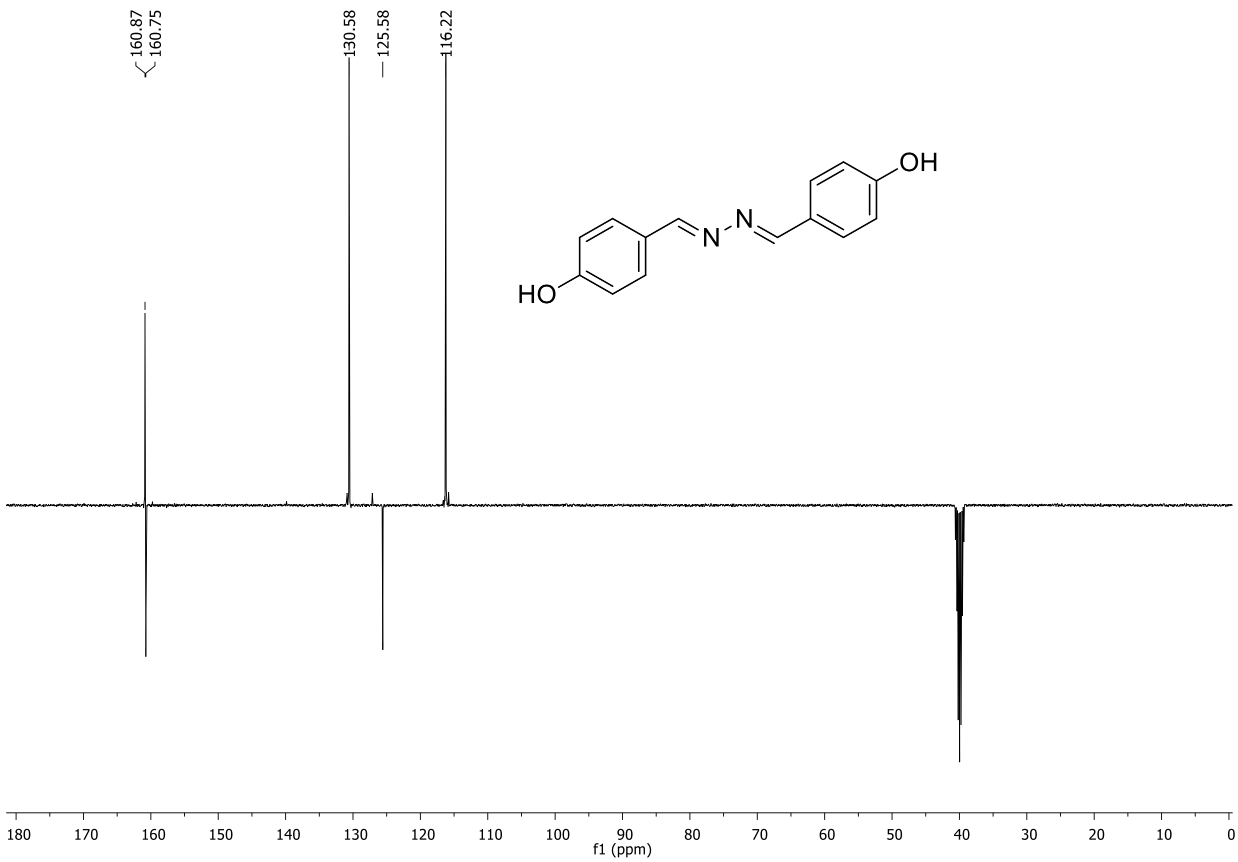

Figure S2. ^{13}C-NMR of 4,4'-((1E,1'E)-hydrazine-1,2-diylidenebis(methanylylidene))diphenol.

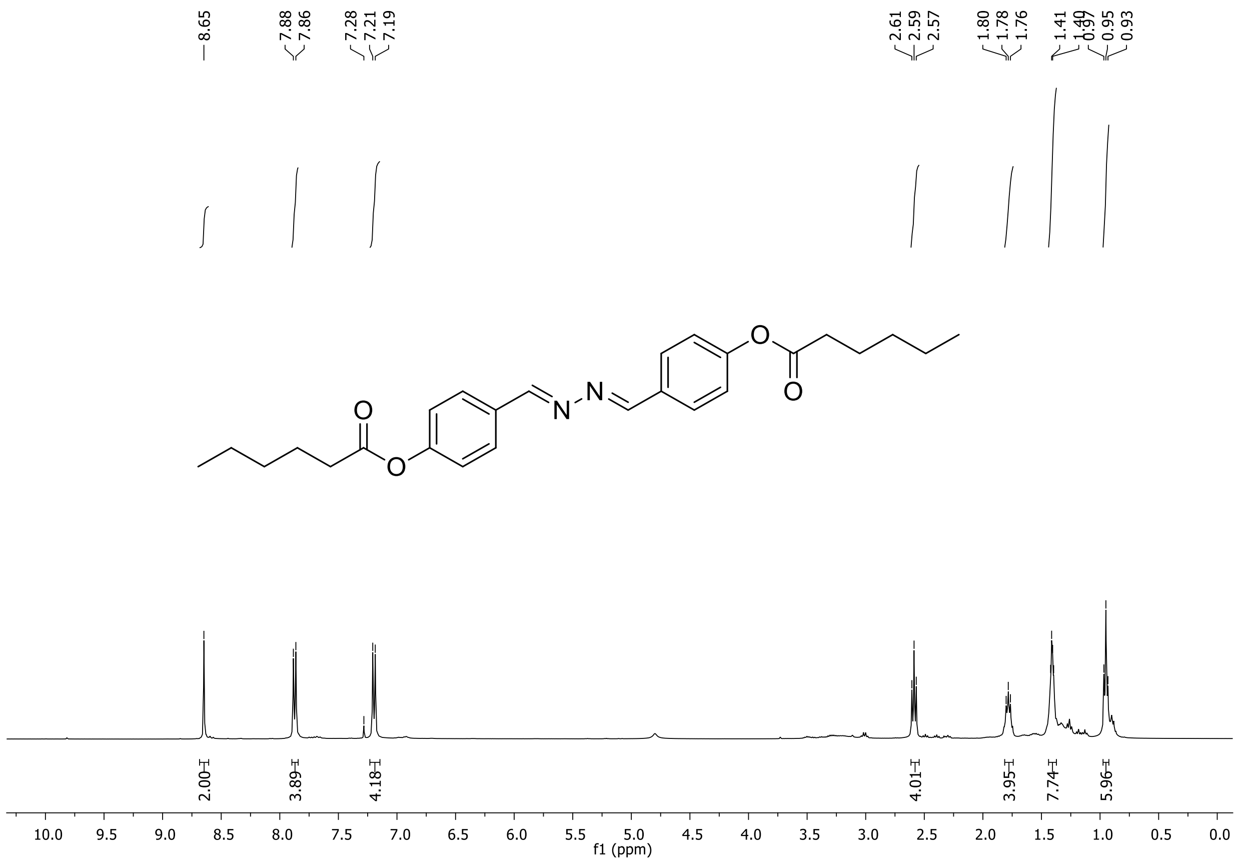

Figure S3. ^{1}H-NMR of ((1E,1'E)-hydrazine-1,2-diylidenebis(methanylylidene))bis(4,1-phenylene) dihexanoate.

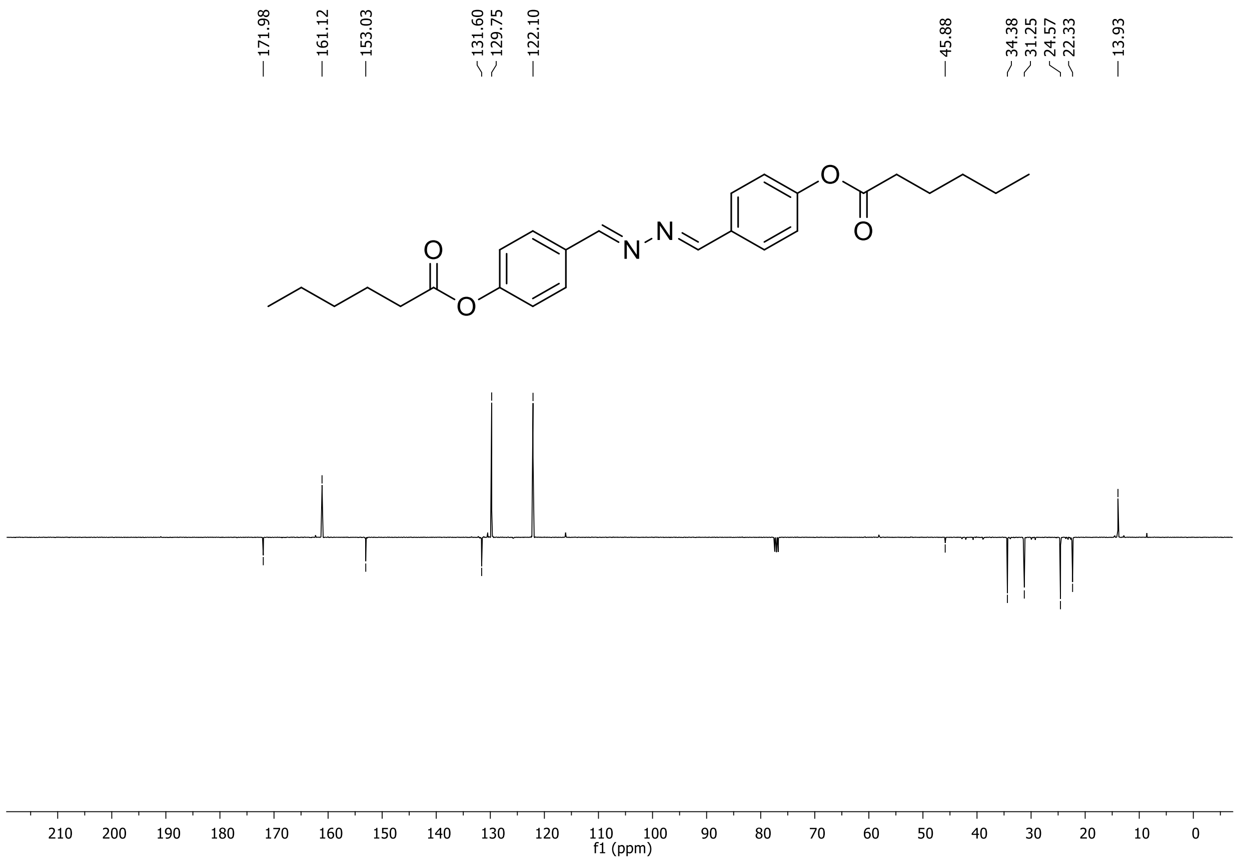

Figure S4. ^{13}C-NMR of ((1E,1'E)-hydrazine-1,2-diylidenebis(methanylylidene))bis(4,1-phenylene) dihexanoate.

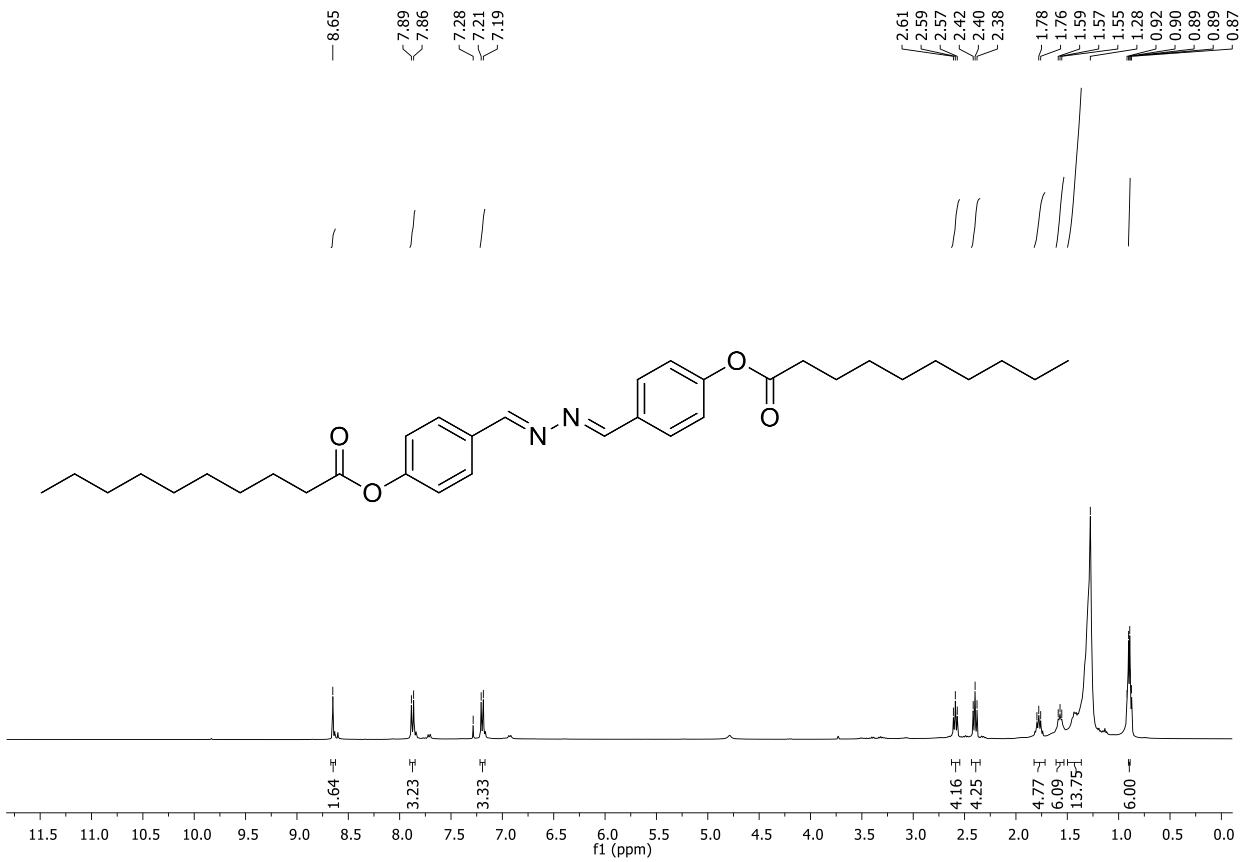

Figure S5. ^{1}H-NMR of ((1E,1'E)-hydrazine-1,2-diylidenebis(methanylylidene))bis(4,1-phenylene) bis(decanoate).

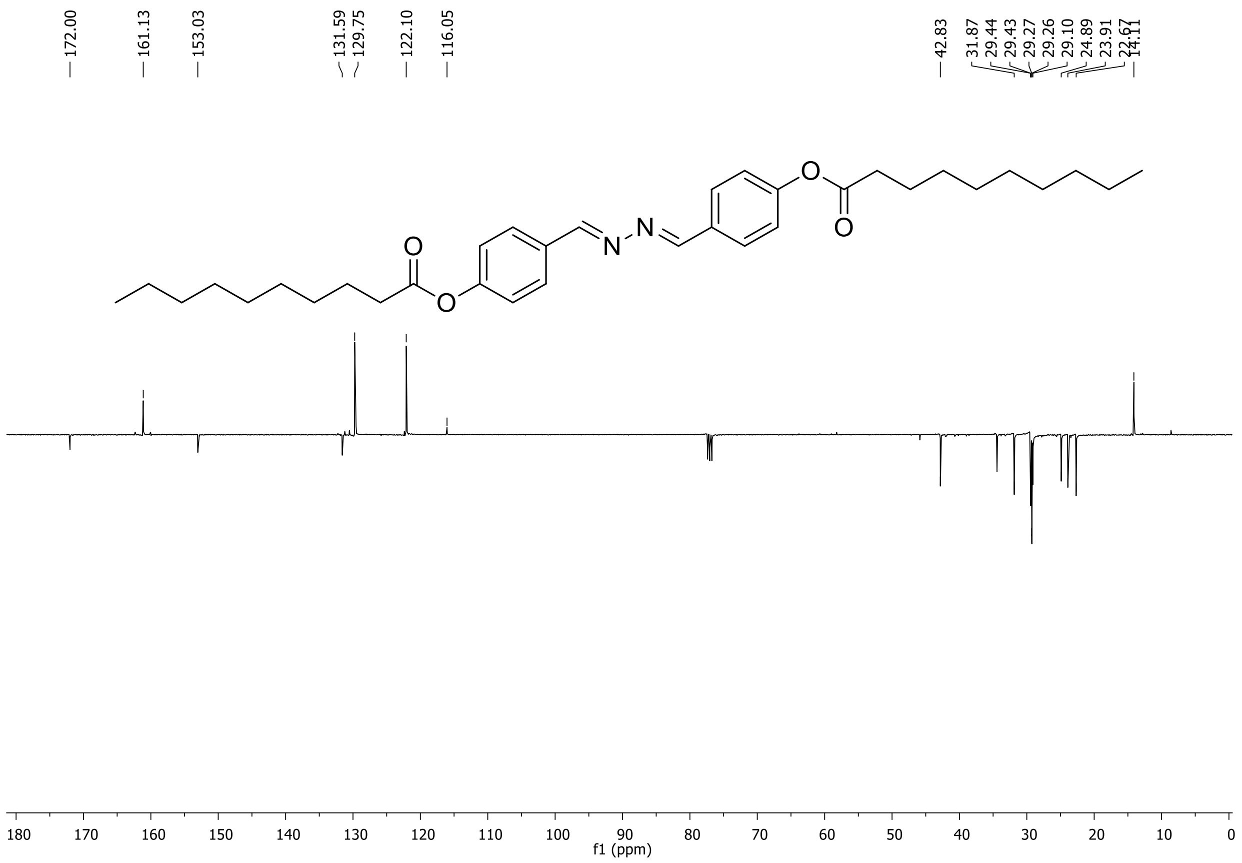

Figure S6. ^{13}C-NMR of ((1E,1'E)-hydrazine-1,2-diylidenebis(methanylylidene))bis(4,1-phenylene) bis(decanoate).

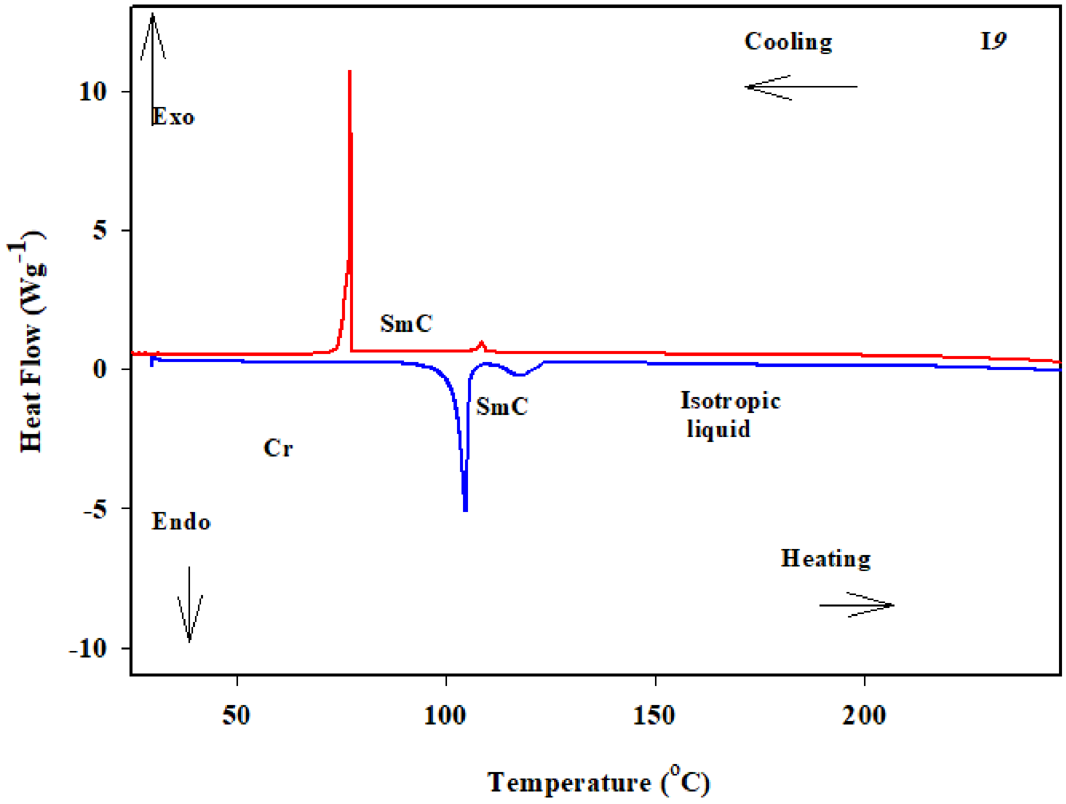

Figure S7. DSC cycles recorded from the second heating and cooling scan with heating rate 10°C/min of compound **I*9***.

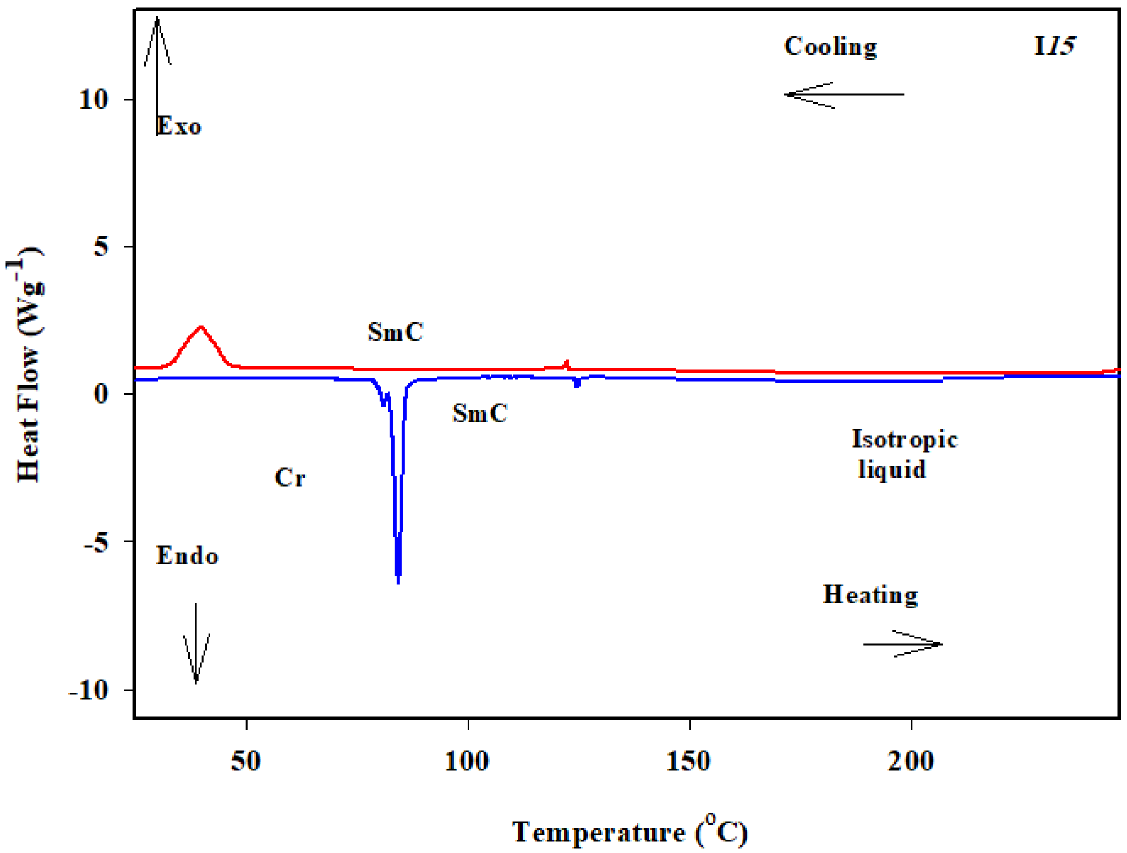

Figure S8. DSC cycles recorded from the second heating and cooling scan with heating rate 10°C/min of compound **I*15***.

6. Optimized geometrical structure of In derivatives with atom numbering

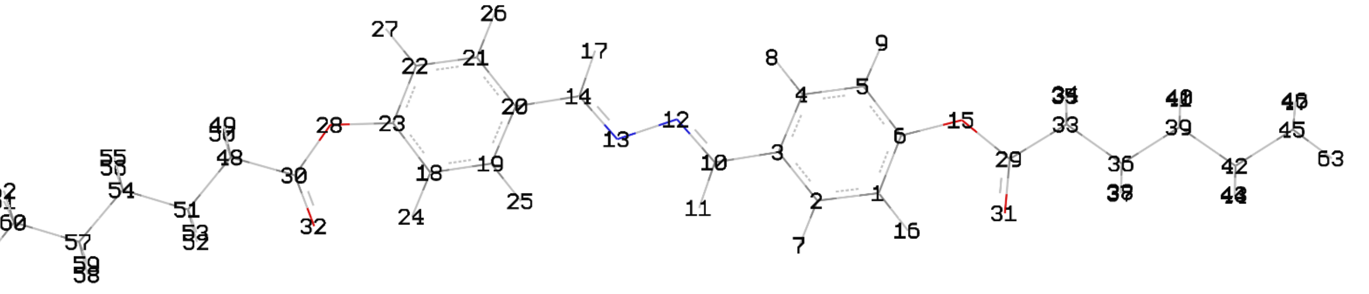

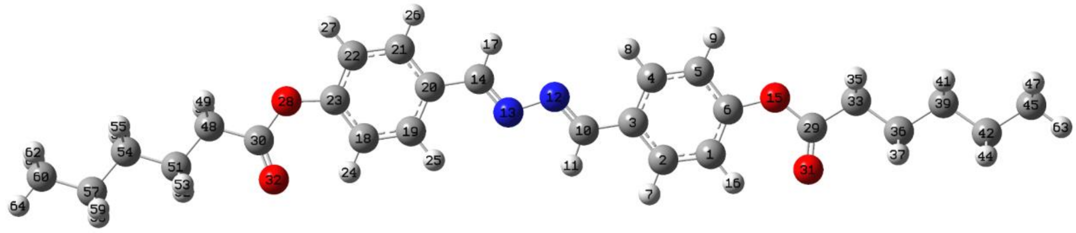

Dihedral	Angle	Bond	NC	NB	NA	Symbol	Tag
						C	1
		1.3966194			1	C	2
	121.7091586	1.4045293		1	2	C	3
-0.0031870	118.5775241	1.4106606	1	2	3	C	4
-0.0071474	120.3927458	1.3862245	2	3	4	C	5
0.0078579	118.2764390	1.3964221	3	2	1	C	6
-179.9886906	118.8246388	1.0826139	6	1	2	H	7
-179.9786508	118.7912717	1.0798997	2	3	4	H	8
-179.9828726	121.5328186	1.0799608	3	4	5	H	9
-179.9522414	119.5615235	1.4580159	1	2	3	C	10
0.2343444	118.1103784	1.0891330	2	3	10	H	11
-179.7870855	122.2051203	1.2957630	2	3	10	N	12
-179.9152521	112.8722999	1.4233617	3	10	12	N	13
-179.7012349	112.8737544	1.2955344	10	12	13	C	14
-179.9622179	125.5230927	1.4166022	2	1	6	O	15
-179.9768791	120.6403512	1.0749384	5	6	1	H	16
0.0610689	119.6271819	1.0893695	12	13	14	H	17
-179.8506455	104.1829021	3.7851091	12	13	14	C	18
0.0418624	18.7497830	1.3928161	13	14	18	C	19
-0.0651611	121.4062411	1.4070842	14	18	19	C	20
-0.0406220	118.5589117	1.4081010	18	19	20	C	21
0.0069668	120.7204108	1.3899870	19	20	21	C	22
0.0314301	119.4970471	1.3965712	20	21	22	C	23
0.4605539	139.7266001	1.0750402	13	14	18	H	24
179.8935319	120.0029939	1.0800824	14	18	19	H	25
-179.9970909	119.6815432	1.0825128	19	20	21	H	26

-179.9694732	121.6437628	1.0797811	20	21	22		H	27
-179.8363828	113.3498830	1.4166771	21	22	23		O	28
-0.1717354	126.6699231	1.3928913	1	6	15		C	29
-177.7459515	126.7304272	1.3934335	22	23	28		C	30
-0.2063878	124.0162492	1.2282106	6	15	29		O	31
0.3059918	124.0574336	1.2280269	23	28	30		O	32
179.7263608	109.5495690	1.5050799	6	15	29		C	33
-57.6445760	107.7710481	1.0943446	15	29	33		H	34
55.7650320	107.8577915	1.0940614	15	29	33		H	35
179.1412433	113.3991498	1.5331018	15	29	33		C	36
-58.1952368	109.3336726	1.0932667	29	33	36		H	37
57.3622147	109.3300552	1.0931472	29	33	36		H	38
179.5909179	112.4586805	1.5374057	29	33	36		C	39
-58.1113490	109.4951539	1.0966432	33	36	39		H	40
57.9616077	109.5250175	1.0966310	33	36	39		H	41
179.9380233	113.1290340	1.5382666	33	42	C		39	36
57.7144517	109.1062251	1.0952189	36	39	43	H		42
-57.8805188	109.1107596	1.0952138	36	39	44	H		42
179.9193980	113.1741888	1.5356958	36	39	45	C		42
-59.9614263	111.1455256	1.0929956	39	42	46	H		45
59.9197315	111.1457512	1.0929977	39	42	47	H		45
-179.4502088	109.4721199	1.5051355	23	28	48	C		30
-50.7047622	107.9614947	1.0933876	28	30	49	H		48
62.7227970	107.6345488	1.0949987	28	30	50	H		48
-174.3582688	113.4604135	1.5331963	28	30	51	C		48
-56.6014994	109.3581103	1.0929120	30	48	52	H		51
58.9860889	109.3148966	1.0935111	30	48	53	H		51

-178.9136511	112.3964394	1.5373725	30	48	54	C	51
-57.3334174	109.5149728	1.0966969	48	51	55	H	54
58.7140904	109.4801151	1.0966485	48	51	56	H	54
-179.2977404	113.1796006	1.5382120	48	51	57	C	54
-57.7343277	109.1312844	1.0952441	51	54	58	H	57
57.8647765	109.0976924	1.0952182	51	54	59	H	57
-179.9497556	113.1594181	1.5356699	51	54	60	C	57
59.9365214	111.1470014	1.0929949	54	57	61	H	60
-59.9398069	111.1443109	1.0930276	54	57	62	H	60
179.9799186	111.3125704	1.0918133	39	42	63	H	45
179.9938624	111.3139514	1.0918015	54	57	64	H	60

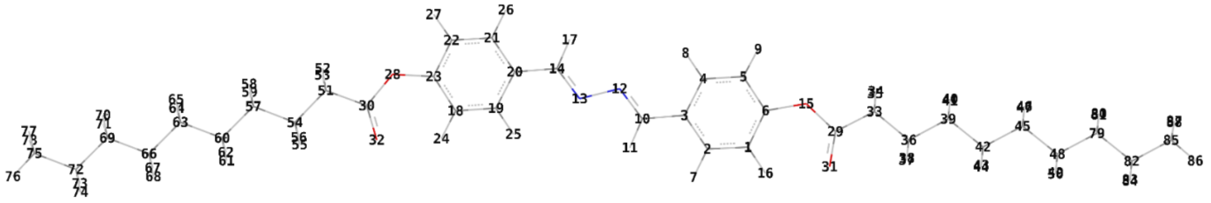

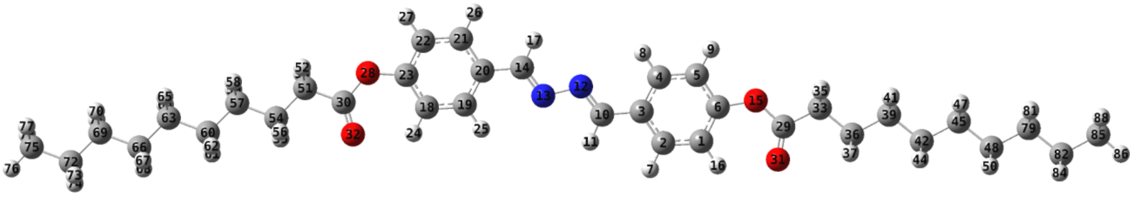

Angle	Bond	NC	NB	NA	Symbol Dihedral	Tag
					C	1
	1.3966592			1	C	2

1.4045304		1	2	C	3
				121.7110729	
1.4106600	1	2	3	C	4
			-0.0248376	118.5763596	
1.3862071	2	3	4	C	5
			0.0156822	120.3912076	
1.3963883	3	2	1	C	6
			0.0052369	118.2748328	
1.0826192	6	1	2	H	7
			179.9874894	118.8277205	
1.0798958	2	3	4	H	8
			-179.9672761	118.7985806	
1.0799680	3	4	5	H	9
			179.9965837	121.5345246	
1.4580307	1	2	3	C	10
			179.9764421	119.5801388	
1.0891358	2	3	10	H	11
			0.2782229	118.1232934	
1.2957443	2	3	10	N	12
			-179.7037538	122.1840955	
1.4233891	3	10	12	N	13
			-179.9835915	112.8925088	
1.2955493	10	12	13	C	14
			-179.5396516	112.8535955	
1.4164574	2	1	6	O	15
			-179.8452165	125.5254414	
1.0749444	5	6	1	H	16
			-179.9393348	120.6431780	
1.0893708	12	13	14	H	17
			0.0279063	119.6168034	
3.7851191	12	13	14	C	18
			-179.9338911	104.2058967	
1.3928382	13	14	18	C	19
			0.2377507	18.7563773	

1.4070844	14	18	19	C	20
			0.0090650	121.4015229	
1.4081018	18	19	20	C	21
			-0.0228484	118.5572375	
1.3899803	19	20	21	C	22
			-0.0285912	120.7255968	
1.3965455	20	21	22	C	23
			0.0528901	119.4959511	
1.0750727	13	14	18	H	24
			0.6933319	139.7130345	
1.0800878	14	18	19	H	25
			179.9870960	119.9957460	
1.0825174	19	20	21	H	26
			179.9905120	119.6732103	
1.0797742	20	21	22	H	27
			-179.9171343	121.6423091	
1.4166505	21	22	23	O	28
			-179.6077708	113.3520813	
1.3930088	1	6	15	C	29
			-0.5615891	126.6904738	
1.3934917	22	23	28	C	30
			-176.4233872	126.7101882	
1.2281969	6	15	29	O	31
			-0.4694964	124.0166628	
1.2279968	23	28	30	O	32
			0.5901393	124.0397184	
1.5049911	6	15	29	C	33
			179.4033535	109.5136198	
1.0944630	15	29	33	H	34
			-58.1587664	107.7185009	
1.0940050	15	29	33	H	35
			55.2202543	107.8734487	
1.5330631	15	29	33	C	36
			178.6748589	113.4629825	

1.0932943	29	33	36	H	37		
		-58.6557345	109.3542761				
1.0930979	29	33	36	H	38		
		56.9296159	109.3348649				
1.5376007	29	33	36	C	39		
		179.1350597	112.3908725				
1.0965521	33	36	39	H	40		
		-58.1828425	109.4133243				
1.0965374	33	36	39	H	41		
		57.8236407	109.4758518				
1.5379544	33	36	39	C	42		
		179.8453750	113.1555998				
1.0961148	36	39	42	H	43		
		57.5437043	109.1973483				
1.0960991	36	39	42	H	44		
		-58.1826580	109.2071275				
1.5381012	36	39	42	C	45		
		179.6678368	113.4472264				
1.0964487	39	42	45	H	46		
		-57.9885964	109.2168182				
1.0964611	39	42	45	H	47		
		57.7636214	109.2380560				
1.5381757	39	42	45	C	48		
		179.8937256	113.5221957				
1.0964086	42	45	48	H	49		
		57.7083213	109.2170142				
1.0963889	42	45	48	H	50		
		-58.0179450	109.2192350				
1.5051112	23	28	30	C	51		
		-179.0577404	109.4922051				
1.0931512	28	30	51	H	52		
		-49.3889298	108.0343763				
1.0951997	28	30	51	H	53		
		64.0670232	107.5712042				

1.5332542	28	30	51	C		54
		-173.1462031		113.4456812		
1.0928006	30	51	54	H		55
		-55.8059244		109.3525535		
1.0935992	30	51	54	H		56
		59.7841302		109.3076299		
1.5376023	30	51	54	C		57
		-178.1200859		112.3931582		
1.0965566	51	54	57	H		58
		-57.0677638		109.4885017		
1.0965516	51	54	57	H		59
		58.9410594		109.4023590		
1.5379369	51	54	57	C		60
		-179.0702894		113.1571630		
1.0960904	54	57	60	H		61
		-57.4119064		109.2138511		
1.0961055	54	57	60	H		62
		58.3111370		109.1834686		
1.5381167	54	57	60	C		63
		-179.5623695		113.4466564		
1.0964288	57	60	63	H		64
		58.2875671		109.2323787		
1.0964819	57	60	63	H		65
		-57.4687652		109.2276814		
1.5381734	57	60	63	C		66
		-179.5748368		113.5238138		
1.0963823	60	63	66	H		67
		58.0166491		109.2046161		
1.0963931	60	63	66	H		68
		-57.7077712		109.2239278		
1.5380185	60	63	66	C		69
		-179.8481670		113.5367100		
1.0966126	63	66	69	H		70
		-57.7004512		109.2593535		

1.0965791 63 66 69 H 71
58.0578264 109.2680351

1.5383751 63 66 69 C 72
-179.8075105 113.5946777

1.0955284 66 69 72 H 73
57.7813517 109.1232938

1.0955716 66 69 72 H 74
-57.8267818 109.1426711

1.5359639 66 69 72 C 75
179.9823258 113.2416636

1.0920279 69 72 75 H 76
179.9262345 111.3983990

1.0930589 69 72 75 H 77
-59.9604498 111.1128350

1.0930682 69 72 75 H 78
59.8239900 111.1028055

1.5380086 42 45 48 C 79
179.8403987 113.5397546

1.0965922 45 48 79 H 80
-57.9472440 109.2579991

1.0966068 45 48 79 H 81
57.8091837 109.2666192

1.5383677 45 48 79 C 82
179.9311206 113.5869899

1.0955644 48 79 82 H 83
57.7572761 109.1372026

1.0955482 48 79 82 H 84
-57.8496359 109.1354421

1.5359556 48 79 82 C 85
179.9542179 113.2511847

1.0920344 79 82 85 H 86
179.9993193 111.3926418

1.0930491 79 82 85 H 87
-59.8990947 111.1109506

1.0930535	79	82	85	H	88
		59.8962779	111.1132502		

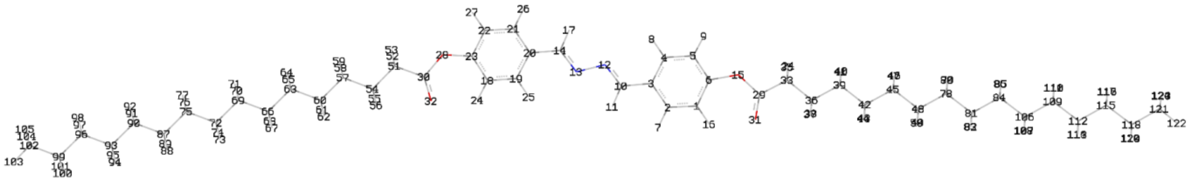

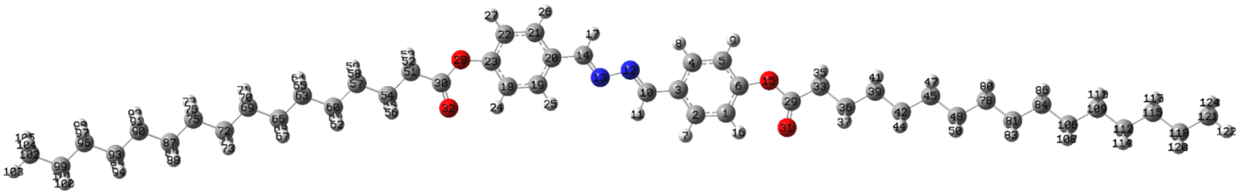

Angle	Bond	NC	NB	NA	symbol	Dihedral	Tag
					C		1
	1.3967864			1	C		2
121.7041385	1.4045457		1	2	C		3
118.5767163	1.4106551	1	2	3	C	0.1045446	4
120.3955879	1.3861212	2	3	4	C	-0.0720628	5
118.2752561	1.3963293	3	2	1	C	-0.0296376	6
118.8334989	1.0826318	6	1	2	H	-179.9493910	7
118.7946901	1.0798794	2	3	4	H	-179.9958881	8
121.5372877	1.0799650	3	4	5	H	-179.9923268	9

119.6375323	1.4581351	1	2	3	C	-179.7121061	10
118.1641214	1.0891417	2	3	10	H	-0.2235688	11
122.1096987	1.2956870	2	3	10	N	179.5975725	12
112.9683999	1.4234191	3	10	12	N	-179.7035576	13
112.7824928	1.2956033	10	12	13	C	179.2420510	14
125.5359147	1.4162534	2	1	6	O	-179.8587055	15
120.6468577	1.0749841	5	6	1	H	-179.8830891	16
119.5832186	1.0893793	12	13	14	H	0.1044325	17
104.2971534	3.7854669	12	13	14	C	-179.6506128	18
18.7704810	1.3929754	13	14	18	C	-0.9784610	19
121.4054696	1.4070350	14	18	19	C	-0.3815721	20
118.5558702	1.4081544	18	19	20	C	-0.1397037	21
120.7254009	1.3898622	19	20	21	C	0.1834273	22
119.5024255	1.3965677	20	21	22	C	-0.0319913	23
139.7196444	1.0751005	13	14	18	H	-0.5084276	24
119.9817020	1.0801017	14	18	19	H	179.5476659	25
119.6652985	1.0825245	19	20	21	H	-179.9454554	26

121.6424357	1.0797572	20	21	22	H	179.8567213	27
113.3000363	1.4165089	21	22	23	O	179.6626732	28
126.7394303	1.3930970	1	6	15	C	-2.3570999	29
126.7921479	1.3933432	22	23	28	C	-179.7636783	30
124.0277949	1.2281535	6	15	29	O	-0.1464662	31
124.0573208	1.2279268	23	28	30	O	-0.9791957	32
109.4373629	1.5049092	6	15	29	C	179.6731181	33
107.6451745	1.0947000	15	29	33	H	-59.4480368	34
107.8797820	1.0938191	15	29	33	H	53.8758806	35
113.5863830	1.5330153	15	29	33	C	177.4201374	36
109.3627650	1.0933821	29	33	36	H	-58.8583686	37
109.3799926	1.0930242	29	33	36	H	56.7833208	38
112.2667828	1.5376020	29	33	36	C	179.0017099	39
109.3730630	1.0966031	33	36	39	H	-58.3780746	40
109.4484400	1.0965495	33	36	39	H	57.5659293	41
113.2904453	1.5379064	33	36	39	C	179.6133015	42
109.2349793	1.0960878	36	39	42	H	57.4893476	43

109.2300728	1.0961205	36	39	42	H<br>-58.2847516	44
113.3344527	1.5380916	36	39	42	C<br>179.6004668	45
109.1813410	1.0965035	39	42	45	H<br>-58.0971482	46
109.2165894	1.0964825	39	42	45	H<br>57.6026140	47
113.6345334	1.5381100	39	42	45	C<br>179.7627939	48
109.2491446	1.0963669	42	45	48	H<br>57.5941752	49
109.2462724	1.0963873	42	45	48	H<br>-58.1771633	50
109.4604243	1.5051204	23	28	30	C<br>178.8791021	51
107.7065457	1.0944564	28	30	51	H<br>-58.2658926	52
107.8843694	1.0939658	28	30	51	H<br>55.1326915	53
113.4641582	1.5330364	28	30	51	C<br>178.6118797	54
109.3568885	1.0933032	30	51	54	H<br>-58.9634934	55
109.3233600	1.0930629	30	51	54	H<br>56.6213234	56
112.3873136	1.5376667	30	51	54	C<br>178.8054826	57
109.4078029	1.0965498	51	54	57	H<br>-58.1338367	58
109.4812157	1.0965283	51	54	57	H<br>57.8766165	59
113.1682212	1.5379279	51	54	57	C<br>179.9195766	60

109.2099893	1.0960751	54	57	60	H -58.3197425		61
109.1866092	1.0960883	54	57	60	H 57.4093111		62
113.4431552	1.5381522	54	57	60	C 179.5043108		63
109.2385777	1.0964547	57	60	63	H 57.8576233		64
109.2126528	1.0964587	57	60	63	H -57.8955765		65
113.5373771	1.5381178	57	60	63	C -179.9963489		66
109.2127605	1.0963649	60	63	66	H 57.6171278		67
109.2239250	1.0963488	60	63	66	H -58.1173617		68
113.5188829	1.5382920	60	63	66	C 179.7234851		69
109.2040610	1.0964640	63	66	69	H -57.8777261		70
109.2104845	1.0964661	63	66	69	H 57.8395481		71
113.5714953	1.5381334	63	66	69	C 179.9887752		72
109.2126055	1.0964258	66	69	72	H 57.7523844		73
109.2136915	1.0964161	66	69	72	H -57.9690275		74
113.5527148	1.5383085	66	69	72	C 179.8793664		75
109.2089670	1.0964644	69	72	75	H -57.8877852		76
109.2004275	1.0964686	69	72	75	H 57.8225727		77

113.4287522	1.5382329	42	45	48	C 179.7030435	78
109.1699430	1.0965121	45	48	78	H -57.9811364	79
109.2017646	1.0964854	45	48	78	H 57.6919336	80
113.6477383	1.5381269	45	48	78	C 179.8689846	81
109.2364741	1.0964321	48	78	81	H 57.6443266	82
109.2392176	1.0964502	48	78	81	H -58.1061154	83
113.4807835	1.5382560	48	78	81	C 179.7617994	84
109.1737515	1.0965126	78	81	84	H -57.9036422	85
109.2022217	1.0964828	78	81	84	H 57.7711822	86
113.5740338	1.5381504	69	72	75	C 179.9642605	87
109.2126197	1.0964452	72	75	87	H 57.8587247	88
109.2067628	1.0964391	72	75	87	H -57.8561947	89
113.5670787	1.5383139	72	75	87	C 179.9985325	90
109.2137020	1.0964518	75	87	90	H -57.8861903	91
109.1960587	1.0964581	75	87	90	H 57.8233699	92
113.5759699	1.5381968	75	87	90	C 179.9600900	93
109.2084636	1.0964716	87	90	93	H 57.9283596	94

109.2017172	1.0964658	87	90	93	H	-57.7812554	95
113.5828545	1.5380719	87	90	93	C	-179.9269052	96
109.2634533	1.0965912	90	93	96	H	-57.8604214	97
109.2469429	1.0965926	90	93	96	H	57.8824342	98
113.6224489	1.5383768	90	93	96	C	-179.9948204	99
109.1363498	1.0955832	93	96	99	H	57.9222587	100
109.1321100	1.0955446	93	96	99	H	-57.6910673	101
113.2447412	1.5360381	93	96	99	C	-179.8946227	102
111.4189009	1.0920742	96	99	102	H	-179.8117447	103
111.0899201	1.0930954	96	99	102	H	-59.6966665	104
111.1029401	1.0930557	96	99	102	H	60.0639591	105
113.6292347	1.5381363	78	81	84	C	179.9492126	106
109.2262525	1.0964555	81	84	106	H	57.6876253	107
109.2323796	1.0964736	81	84	106	H	-58.0474211	108
113.5171218	1.5382718	81	84	106	C	179.8126413	109
109.1818074	1.0964965	84	106	109	H	-57.8571438	110
109.2055566	1.0964693	84	106	109	H	57.8271983	111

113.6049787	1.5381706	84	106	109	C 179.9994200	112
109.2149692	1.0964841	106	109	112	H 57.7321054	113
109.2210810	1.0965037	106	109	112	H -57.9857825	114
113.5626137	1.5380447	106	109	112	C 179.8688257	115
109.2432025	1.0966249	109	112	115	H -57.8602740	116
109.2595380	1.0966001	109	112	115	H 57.8720983	117
113.6155543	1.5383510	109	112	115	C -179.9838093	118
109.1333921	1.0955824	112	115	118	H 57.7455212	119
109.1395450	1.0956028	112	115	118	H -57.8575240	120
113.2619023	1.5359938	112	115	118	C 179.9471829	121
111.4049687	1.0920810	115	118	121	H 179.9532475	122
111.1081905	1.0930629	115	118	121	H -59.9367957	123
111.1047498	1.0930616	115	118	121	H 59.8430866	124

References:

1. Frisch, M.; Trucks, G.; Schlegel, H.B.; Scuseria, G.; Robb, M.; Cheeseman, J.; Scalmani, G.; Barone, V.; Mennucci, B.; Petersson, G. Gaussian 09, revision a. 02, gaussian. *Inc., Wallingford, CT* **2009**, *200*.
2. Dennington, R.; Keith, T.; Millam, J. GaussView, version 5. **2009**.
